# Feasibility of a Sleep Self-Management Intervention in Pregnancy Using a Personalized Health Monitoring Device: Protocol for a Pilot Randomized Controlled Trial

**DOI:** 10.2196/12455

**Published:** 2019-05-29

**Authors:** Marquis Hawkins, Favorite Iradukunda, Mary Paterno

**Affiliations:** 1 Department of Biostatistics and Epidemiology University of Massachusetts Amherst Amherst, MA United States; 2 Department of Epidemiology University of Pittsburgh Pittsburgh, PA United States; 3 College of Nursing University of Massachusetts Amherst Amherst, MA United States

**Keywords:** eHealth, pregnancy, personal health monitoring, behavior, maternal health

## Abstract

**Background:**

Sleep disruptions are common during pregnancy and associated with increased risk of adverse maternal outcomes such as preeclampsia, gestational diabetes, prolonged labor, and cesarean birth. Given the morbidity associated with poor sleep, cost-effective approaches to improving sleep that can be disseminated in community or clinical settings are needed. Personal health monitor (PHM) devices offer an opportunity to promote behavior change, but their acceptability and efficacy at improving sleep in pregnant women are unknown.

**Objective:**

The goal of the paper is to describe the protocol for an ongoing pilot randomized controlled trial that aims to establish the feasibility, acceptability, and preliminary efficacy of using a PHM device (Shine 2, Misfit) to promote sleep during pregnancy.

**Methods:**

The proposed pilot study is a 12-week, parallel arm, randomized controlled trial. Pregnant women, at 24 weeks gestation, will be randomized at a 1:1 ratio to a 12-week sleep education plus PHM device group or a sleep education alone comparison group. The primary outcomes will be measures of feasibility (ie, recruitment, enrollment, adherence) and acceptability (ie, participant satisfaction). The secondary outcomes will be self-reported sleep quality and duration, excessive daytime sleepiness, fatigue, and depressive symptoms.

**Results:**

Recruitment for this study began in September 2017 and ended in March 2018. Data collection for the primary and secondary aims was completed in August 2018. We anticipate that the data analysis for primary and secondary aims will be completed by December 2019. The results from this trial will inform the development of a larger National Institutes of Health grant application to test the efficacy of an enhanced version of the sleep intervention that we plan to submit in the year 2020.

**Conclusions:**

This study will be the first to apply a PHM device as a tool for promoting self-management of sleep among pregnant women. PHM devices have the potential to facilitate behavioral interventions because they include theory-driven, self-regulatory techniques such as behavioral self-monitoring. The results of the study will inform the development of a sleep health intervention for pregnant women.

**Trial Registration:**

ClinicalTrials.gov NCT03783663; https://clinicaltrials.gov/ct2/show/NCT03783663 (Archived by WebCite at http://www.webcitation.org/779Ou8hon)

**International Registered Report Identifier (IRRID):**

DERR1-10.2196/12455

## Introduction

Sleep disruptions are common during pregnancy [1] and are associated with an increased risk of adverse maternal/fetal outcomes such as hyperglycemia [2,3], gestational diabetes [4], preeclampsia [5], cesarean delivery [6], longer labor [7], and delivering a low birthweight infant [8]. Additionally, women with poor sleep in pregnancy experience increased depressive symptoms during pregnancy [9] and postpartum depression [10], which increase their risk of depression later in life [11].

While sleep disruptions among pregnant women are often due to physical (eg, pain, nocturia, growing fetus) and hormonal changes [12], lifestyle factors such as sleep hygiene (ie, behavioral and environmental habits that promote or disrupt sleep) also contribute to sleep disruptions. For example, in a convenience sample of 197 pregnant women in their third trimester of pregnancy in Taiwan, poor sleepers were shown to have worse sleep hygiene than good sleepers [13]. Moreover, other health behaviors, such as physical activity and diet, are associated with better sleep duration in pregnancy [14-17]. These studies demonstrate that modifiable factors contribute to sleep disruptions during pregnancy, suggesting that interventions could make a positive impact on sleep in this group. A review of seven nonpharmacological (eg, acupuncture, physical activity, massage) sleep interventions during pregnancy showed trends of improving sleep; however, the studies have generally been of low quality and none focused on sleep hygiene [18]. Given the scarcity of effective approaches to promote sleep among pregnant women, there is a need to develop interventions that can be easily disseminated in community or clinical settings.

Personal health monitors (PHMs) have grown in popularity, with an estimated 1 in 8 consumers now owning a PHM [19]. The popularity of PHM use may provide an opportunity to promote positive health behaviors, such as sleep health, on a large scale. Many PHM devices include theory-driven behavior change techniques such as self-monitoring, goal setting, review of behavioral goals, rewards, and facilitation of comparisons with peers [20]. Several researchers have incorporated the use of PHMs into standard interventions to promote physical activity and weight loss [21-26]. However, there is limited evidence on the efficacy of wearable devices to promote sleep.

Here we describe the protocol for an ongoing pilot randomized controlled trial [NCT03783663] that aims to establish the feasibility, acceptability, and preliminary efficacy of using a PHM device (Shine 2, Misfit) to promote sleep during pregnancy. Specifically, the primary aim of the study will be to (1) establish the feasibility and acceptability of conducting a 12-week intervention for sleep self-management among pregnant women using a PHM device and (2) determine the feasibility of collecting data on sleep and physical activity using a PHM device. The secondary aim will be to determine the preliminary efficacy of the trial on improving self-reported sleep quality and nocturnal sleep duration and decreasing sleep disturbances, excessive daytime sleepiness, fatigue, and depressive symptoms.

## Methods

### Study Design

**Figure 1:**
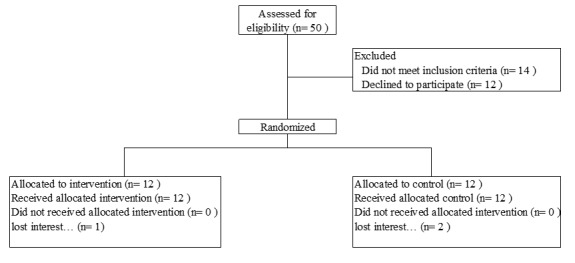
Participant flowchart.

This pilot study is a 12-week, parallel arm, randomized controlled trial. Participants will be randomized at a 1:1 ratio to a 12-week sleep education plus PHM device group or a sleep education alone comparison group at approximately 24 weeks gestation of pregnancy (Figure 1). This time period was chosen to capture data during the third trimester of pregnancy when sleep may worsen [1] while allowing sufficient time for study completion prior to birth. We will use blocked randomization to ensure equal group size due to the small pilot sample size. The study has been approved by the institutional review board of the University of Massachusetts Amherst. All participants will be required to provide informed written consent prior to enrollment.

### Procedures

At the time of recruitment (t_0_) (14 to 24 weeks gestation), participants will complete a screening survey to determine their eligibility. At 24 weeks gestation (t_1_), eligible participants will enroll in the study and complete a baseline survey to assess demographic characteristics and secondary outcome measures. Both researchers and participants will be blinded to group assignment at the baseline assessment and during the educational intervention. All participants will receive an individual 30-minute educational intervention on sleep hygiene during pregnancy delivered by a study registered nurse at enrollment. The focus on the sleep education will be to provide tips on how to achieve a better night’s sleep during pregnancy such as managing pregnancy weight gain with aerobic activity and keeping a consistent schedule. Participants will also receive a sleep hygiene brochure, adapted from a Massachusetts Lung and Allergy sleep clinic sleep hygiene brochure to include information specific to pregnancy. We piloted the development of the brochure through an online survey that asked about the usefulness of the information and overall aesthetics. We used similar recruitment approaches (ie, Facebook) and eligibility criteria to identify women in the brochure development pilot as will be used for the pilot intervention to ensure the feedback is appropriate for the target population. Based on the participant feedback, we made modifications to the brochure’s graphical design; the information content was deemed useful. After completing the baseline survey and sleep hygiene education, participants and researchers will be unblinded to the group assignment (t_2_). Assignments will be made prior to the start of recruitment by randomizing study identification (ID) numbers into intervention or control group using Web-based randomization software [27]. The results of the randomization process will be placed into individually sealed envelopes labeled with study ID numbers. At t_2_, the study nurse will open the envelope for the ID number assigned to the participant and reveal group assignment. Those in the intervention group will then receive the intervention.

At 36 weeks gestation (t_3_), all participants will complete an in-person follow-up survey to assess the same secondary outcome measures as at t_1_. Participants will also complete a brief qualitative interview to determine their satisfaction with the sleep hygiene education and, for those in the intervention group, the self-monitoring intervention. Participants will complete a final follow-up phone survey approximately 2 to 4 weeks after delivery (t_4_).

### Study Population

We will recruit pregnant women who reside within approximately 50 miles of the University of Massachusetts Amherst campus. To be eligible to participate in the study, at the time of recruitment (t_0_) women must (1) be aged 18 years or older, (2) be 14 to <24 completed weeks gestation, (3) have no known maternal or fetal complications, (4) have a mobile phone compatible with the study PHM device, (5) have internet access, (6) be English speaking, and (7) be receiving prenatal care. We will exclude women with preexisting diabetes mellitus, hypertension, or a diagnosed sleep disorder, as some research demonstrated that associations between gestational diabetes and hypertension and a preexisting diagnosed sleep disorder would bias study results [28]. For the purpose of establishing feasibility and acceptability of recruitment and implementing the intervention, the sample size goal is 10 women per group. To account for attrition, we will oversample by 20%, recruiting 12 women per group.

Recruitment will be conducted through advertisements posted (1) at local commercial and community centers frequented by pregnant women in western MA, (2) at a women’s health clinic in western MA, (3) at community centers with diverse memberships such as churches, and (4) on Facebook and Craigslist. The study nurse will also recruit participants in person in the waiting room of the women’s health clinics at the University of Massachusetts Medical Center. During screening/enrollment, we will document the source of recruitment for each participant.

### Intervention

#### Conceptual Framework

The conceptual framework for the intervention is based on a meta-regression of lifestyle (ie, healthy eating and physical activity) interventions among adults by Michie et al [29]. The authors found that lifestyle interventions incorporating self-monitoring and one other self-regulatory technique derived from control theory (ie, intention formation, setting goals, feedback on performance, review of behavioral goals) were more effective at promoting behavior change (pooled effect size = 0.38; 95% CI 0.27 to 0.49) than studies not including these techniques (pooled effect size = 0.27; 95% CI 0.21 to 0.34). Likewise, in their systematic review and meta-analysis on the efficacy of postpartum physical activity interventions, Gilinsky et al [30] found that efficacious interventions were twice as likely to include self-monitoring than nonefficacious interventions. Guided by this conceptual framework, the sleep intervention will include (1) behavioral self-monitoring (using the Shine 2), (2) goal setting (30-minute sleep education session), (3) feedback on performance (Shine 2), and (4) review of behavioral goals (Shine 2).

#### Instrumentation

The intervention group will be given a Shine 2 to monitor sleep throughout the 12-week intervention period. The Shine 2 is a triaxial accelerometer that can be worn on the wrist, waist, neck, pocket, or shoe. The Shine 2 measures steps, intensity, energy expenditure, distance traveled, and sleep duration using a proprietary algorithm. The Shine 2 was selected because it can be worn continuously (even during water-based activities), does not require charging (approximate 6-month battery life), and does not require participants to set the device to sleep mode in order to capture sleep patterns, features that may improve adherence to the intervention. The Shine 2 has been shown to provide equivalent estimates of total sleep time as in-lab polysomnography (*r*=.87), the gold standard for sleep assessment [31].

**Figure 2:**
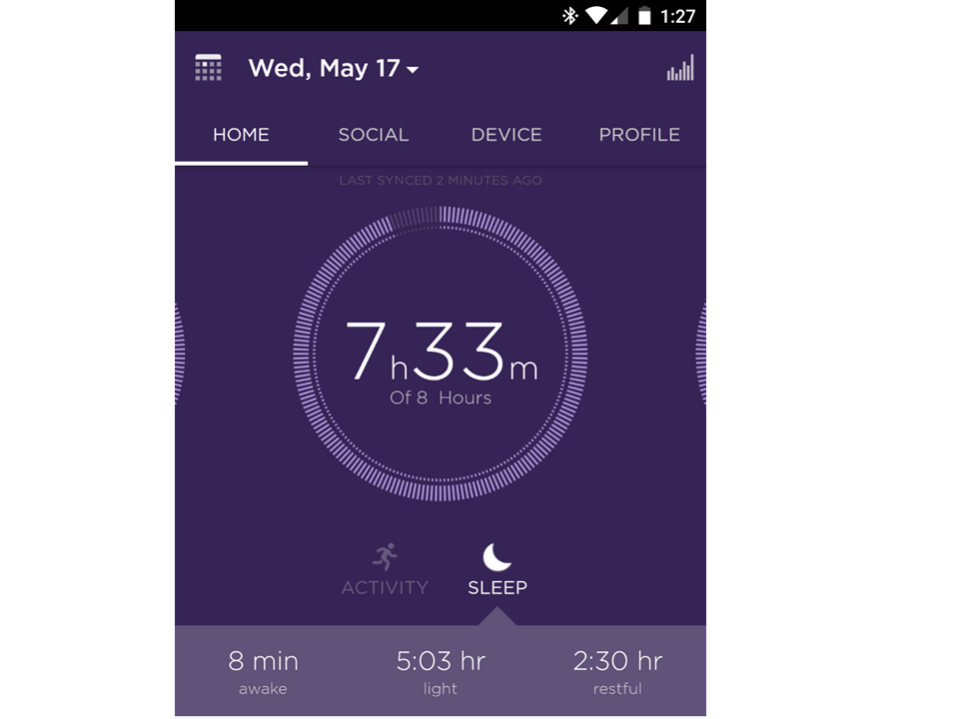
Misfit Shine 2 dashboard displaying the previous night’s sleep duration and self-selected sleep goal.

**Figure 3:**
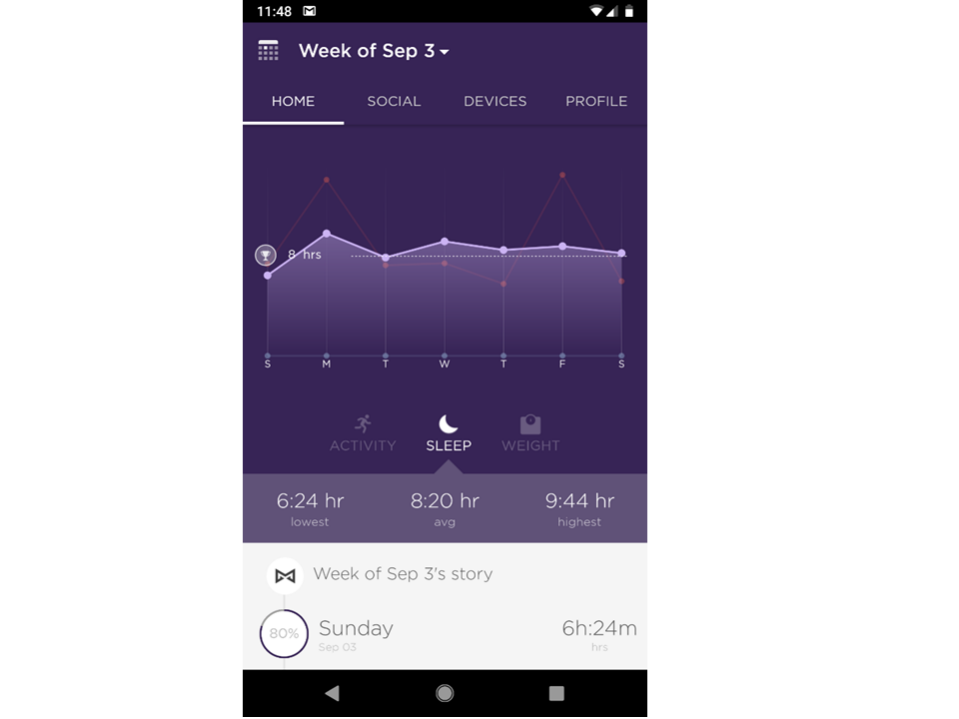
Misfit Shine 2 dashboard displaying weekly trend of sleep duration and self-selected sleep goal.

Participants will be instructed to wear the device on the wrist, which is better for capturing sleep than the other wear locations. Participants will be instructed on how to self-monitor total sleep time and select a goal for that behavior. Figures 2 and 3 show examples of the feedback participants receive on their sleep relative to their self-selected sleep goals. Further, participants will view feedback on their sleep time daily on the Shine 2 mobile phone app, which they can use to monitor their progress toward achieving their behavioral goals. The Shine 2 syncs with iPhone, Android, and Windows phones. Instructions for syncing the Shine 2s to phones will be included in an instructional brochure designed by the study team. Study-specific Gmail accounts and passwords will be created for each study ID so we can access their sleep and physical activity data. Participants will download the Shine 2 app to their mobile phone and create a Shine 2 account using the assigned study Gmail address. Participants will also download the IFTTT app, a free platform that facilitates downloading and sharing data from a PHM [32]. Participants will activate two IFTTT applets that connect Shine 2 with a Google spreadsheet in Google Drive: (1) “Save your Misfit Shine 2 sleep logs to a Google spreadsheet” and (2) “Document your daily activity summaries.” The applets will automatically download the Shine 2 data for sleep daily to the participant’s study Gmail account, making it viewable by the research team. This will allow the research team to view participant progress toward meeting their sleep goals. To enhance data security, the study Gmail account will be used only to collect the Shine 2 data (ie, will not be used for email or other purposes), and the Google Drive accounts will be accessed only by study team members using computers that are password-protected and encrypted. Further, no identifying personal information will be contained within the Gmail account. At the end of the study, participants will be instructed to change their email addresses and passwords in their Shine 2 account to a personal email and password so investigators will no longer have access to their Shine 2 accounts. Once all Shine 2 data have been extracted from the study Google Drive accounts, the accounts will be closed. A team member will conduct follow-up calls to intervention participants one week after enrollment and then monthly to address any issues with using the study device and to answer questions.

#### Intervention Fidelity

The sleep hygiene education session will be provided to both groups by the study nurse. To increase fidelity, a specific protocol for delivering the education to each group will be developed by the principal investigator, and the study nurse will be trained by the principal investigator on the protocol. The delivery of education to study participants will be audio recorded with a digital recorder. The principal investigator will listen to each recording to ensure that participants are receiving the same information per the protocol. In the event that deviation from the protocol is noted, the principal investigator and study nurse will meet to review and retrain on the protocol.

### Primary Outcomes: Intervention, Feasibility, and Acceptability

Table 1 describes the schedule of study assessments for primary and secondary outcomes measures.

#### Recruitment

At t_0_, participants will be asked how they learned about the study (eg, Facebook, community center), with follow-up questions to gather specific details as necessary. The frequency and percentage of participants recruited by each recruitment method will be calculated. We will present descriptive data on the number screened, eligible, and randomized from each source.

#### Retention

Retention may be a challenge due to the longitudinal nature of the study. Each participant will be asked to provide multiple contact methods. Increasing incentives will be built into the study at each data collection time point ($20, $30, and $40 gift cards) to encourage retention for both the intervention and control groups. Participants in the intervention arm will keep the Shine 2 device at the study end. Participants in the control arm will be offered a Shine 2 at the study end. The ability to successfully contact participants, deliver the intervention, and collect data at each time point will be recorded to calculate the percentage of participants retained.

To evaluate participant retention with the intervention protocol, the study team will take two actions. First, we will track sleep data weekly for each participant by viewing their sleep log in the study Gmail account. Then, if a lack of data in the sleep log suggests that the participants are not wearing the monitor daily, we will call the participants to find out why they are not using the device, help them problem solve any issues, and encourage them to continue with study adherence.

**Table 1:** Schedule of study assessments for primary and secondary outcomes.

Characteristics			Assessment			
			Screening/enrollment	Baseline	Active intervention	
			t_0_	t_1_	t_2_	t_3_
**Primary outcomes**						
	**Recruitment/enrollment**					
		Eligibility screen	X			
		Informed consent	X			
		Allocation			X	
		Retention				X
		Participant satisfaction				X
**Secondary outcomes**						
	Sleep quality and duration			X		X
	Sleep disturbances			X		X
	Excessive daytime sleepiness			X		X
	Fatigue			X		X
	Depressive symptoms			X		X

#### Participant Satisfaction

Participants in both groups will be asked to complete a semistructured interview at the 12-week follow-up data collection meeting (t_3_). The interview questions will ask participants about their sleep, barriers and facilitators of sleep, and whether the brief sleep counseling was helpful. Women in the intervention group will additionally be asked about their experience wearing the Shine 2, their likes, dislikes, and whether they believe it would be helpful to other pregnant women.

### Secondary Outcomes

#### Sleep Quality and Duration

The Pittsburgh Sleep Quality Index (PSQI) will be used to measure sleep quality and duration [33]. The PSQI is a 19-item scale measuring perceived sleep quality and disturbance over the past month. For this study, the PSQI will be adapted to measure perceived sleep quality over the past week. The PSQI has been used in numerous studies with pregnant women and has been validated for pregnant women using confirmatory factor analysis and has a reliability of .74 [34]. Scores ≥5 will be considered indicative of poor sleep.

#### Sleep Disturbances

The PROMIS Short Form v1.0–Sleep Disturbance 6a will be used to measure sleep disturbances [35]. This validated 6-item scale measures individual perceptions of sleep quality and disturbance in the past seven days using a 5-point Likert scale. It is scored by summing responses for all items (two are reverse scored); higher scores indicate higher sleep disturbance.

#### Excessive Daytime Sleepiness

The Epworth Sleepiness Scale is an 8-item measure of daytime sleepiness [36]. It has been validated for measuring symptoms of daytime sleepiness in pregnant women using principal components and confirmatory factor analysis and has a reliability coefficient of .75 [37]. Higher cumulative scores indicate higher sleepiness.

#### Fatigue

The PROMIS Fatigue Short Form 4a will be used to measure fatigue. This 4-item scale measures fatigue in the past seven days with a 5-point Likert scale [38]. Responses for all items are summed; higher scores indicate higher fatigue symptoms.

#### Depressive Symptoms

Two scales will be used to measure depressive symptoms in the past seven days. The PROMIS Depression Short Form 6a is a 6-item scale scored by summing responses for all items, with higher scores indicating higher depressive symptoms. It has been validated for use with several chronic illnesses [39]. Although this scale has not been validated in pregnant women, it is a common data element measurement tool that is supported by the National Institutes of Heath. This will allow us to upload deidentified data gathered with this tool to the National Institute for Nursing Research Common Data Elements Repository. We will additionally measure sleep using the Edinburgh Postnatal Depression Scale, a 10-item scale designed to detect depressive symptoms in postpartum women [40], with support for detection of depressive symptoms in pregnancy [41]. Scores range from 0 to 30; a higher score indicates higher depressive symptoms. We will use scores >12 to indicate depressive symptoms.

### Covariates

We will collect information on demographic characteristics and eating habits. Demographic characteristics will include age, ethnic background, racial background, relationship status, level of education, employment status, hours worked per week, and income level. Eating habits will be measured using the Dietary Targets Monitor, a 9-item self-report questionnaire assessing consumption of fruits, vegetables, starchy foods, and other types of foods such as meat and fish [42]. Recommended standard portion for each group will be used to calculate daily, weekly, and monthly consumptions of different foods [42].

### Data Analysis

We will use descriptive statistics to determine the feasibility and acceptability of the pilot intervention. Specifically, we describe recruitment and dropout rates in each group and noncompliance with the intervention among the intervention group only. To assess noncompliance we will use data from the IFTTT applet to determine the proportion of days the monitor was worn during the study period. High compliance will be defined as wearing the monitor at least 80% of days during the study period.

Qualitative methods will be used to determine the acceptability of the intervention, using a qualitative descriptive design [43,44]. The audio recorded interviews will be organized using NVivo 11 (QSR International Inc) software. Recordings will be professionally transcribed verbatim, and transcripts will be checked for accuracy. Descriptive coding will be used to identify and link comparable content and categorize data at a basic level [43]. For further analysis of descriptive codes, content analysis will be used to describe patterns and summarize findings in the data [44]. Memos will be written throughout the analytic process to capture the analysis process and the researchers’ responses to the data. Trustworthiness will be determined through peer debriefing between the research assistant and the principal investigator and the audit trial of documentation of the analysis, including coding, memos, and findings.

To examine the preliminary efficacy of the intervention, all secondary outcome variables will be modeled as continuous outcomes. We will compare baseline characteristics of the intervention groups to ensure randomization was successful. We will present the mean and standard deviation or median and interquartile range for each secondary outcome at baseline and postintervention. A paired *t* test will be used to compare baseline and postintervention means separately in each group. A Student *t* test will then compare the change in each measure between the two intervention groups. Finally, we will calculate within and between-group effect sizes using Cohen d (M_ΔI_ – M_ΔC_ / SD_pooled I and C;_ M_t2_ – M_t3_ / SD_pooled t2 and t3_).

## Results

Recruitment for this study began in September 2017 and ended in March 2018. Data collection for the primary and secondary aims was completed in August 2018. We anticipate that the data analysis for primary aims, evaluating the feasibility and acceptability of the intervention, will be completed by July 2019. We anticipate that the data analysis on the secondary aims, examining the preliminary efficacy of the trial on sleep quality and duration, sleep disturbance, excessive daytime sleepiness, fatigue, and depressive symptoms, will be completed by December 2019. The results from this trial will inform the development of a larger National Institutes of Health grant application to test the efficacy of an enhanced version of the sleep intervention that we plan to submit in the year 2020.

## Discussion

### Principal Findings

This 12-week, parallel arm, pilot randomized controlled trial proposes to establish the feasibility and acceptability of conducting a 12-week intervention for sleep self-management among pregnant women using a PHM device and determine the feasibility of collecting data on sleep and physical activity using a PHM device. Secondarily, the trial will determine the preliminary efficacy of the trial on improving self-reported sleep quality and nocturnal sleep duration and decreasing sleep disturbances, excessive daytime sleepiness, fatigue, and depressive symptoms. This study is novel because it will be the first to apply a PHM device as a tool for promoting self-management of sleep among pregnant women. We anticipate that at least 80% of participants will wear the PHM device daily throughout the study period and report high satisfaction with the intervention. Further, we anticipate that participant feedback will inform the design of a larger randomized controlled trial aiming to improve sleep quality and duration during pregnancy using a PHM device.

Specifically, findings from the pilot study will inform the recruitment and intervention implementation, including strategies to increase adherence. As far as refining our recruitment plan, we will learn important information about the demographic characteristics (eg, race and ethnicity, socioeconomic status, baseline sleep characteristics) of the sample population. Our recruitment goal is for at least 20% of the sample to come from racial and ethnic minority groups. If we fail to recruit a racial and ethnically diverse sample, we will identify specific recruitment barriers and refine our recruitment strategy. Second, we will identify which recruitment approach (eg, Facebook, flyering at local clinics) is the most cost effective. Third, identifying weekly and monthly recruitment yields will allow us to determine if our recruitment strategy could feasibly enroll participants for an adequately powered study. We will need to consider the length of the recruitment period, adding clinical sites, and budgetary considerations for the larger trial.

In addition to recruitment, participant adherence is another critical challenge this feasibility study will help inform. Since previous studies did not use a PHM device specifically to monitor sleep, we would like to first address if pregnant women will wear them for this purpose. Through the IFTTT app, we will be able to identify when the monitor is not worn and elicit feedback from participants on any barriers to wearing the device. If specific benchmarks for retention are not met (>80%), we will refine our current approach (eg, incentive, engagement strategies) to address any challenges that exist.

Last, this feasibility trial will provide important information about the implementation of the intervention. The intervention was meant to be a low-contact intervention to be consistent with the limited time resources in a clinic setting. Through the participant satisfaction interview, we will determine whether the initial counseling session and sleep brochure address challenges specific to pregnant women. Concerning the instruments for the intervention (Shine 2), we will learn if there are specific features participants liked or did not like about the device and the extent to which participants used the device to self-monitor their sleep. Identifying specific features will allow us to be responsive to rapid changes in technology, steering us to select a device that contains features participants find particularly helpful. More generally, we will learn if wearing the PMH device serves as a motivator to participants to monitor their sleep more closely and identify behaviors affecting their sleep.

Given the prevalence and morbidity of sleep disturbances during pregnancy, it is essential to identify cost-effective approaches to promote better sleep in this group. Wearable devices have the potential to facilitate behavioral interventions as they include theory-driven self-regulatory techniques such as behavioral self-monitoring. There has been increasing interest in incorporating PMH devices into clinical settings. For example, PHM devices have been used to monitor inpatient recovery [45]. In an outpatient care setting, PHM devices can be used to track health behaviors remotely and monitor progress toward meeting patient-centered goals around sleep [46,47]. Data from PHM devices can be integrated into electronic medical records. For the patient, tracking behavior more closely can help the patient identify patterns and make choices to change behavior to improve sleep [48]. No prior studies have used a PHM to promote sleep during pregnancy; therefore, novel intervention strategies need to be developed and refined.

### Limitations

This study will, however, have important limitations that warrant discussion. First, the study requires that participants have access to mobile phones compatible with the study monitor; therefore, individuals of low socioeconomic status may potentially be excluded from the study [49]. However, lower income Americans have made gains in technology adoption including the use of mobile phones, which is likely to continue in the future. In addition, since there are multiple components of the intervention (ie, PHM and follow-up calls), we will not be able to discern which component explains differences between the groups, if any are observed. We will keep the follow-up calls briefs and use them primarily as a method of collecting information on the study’s feasibility throughout. However, we won’t be able to remove the potential effects repeated contacts can have on a participant’s ability to change behavior. Third, we will not exclude participants based on their use of any PHM. Therefore, control group participants may have access to a PHM during the study. However, none of the participants will be using our specific study monitor at baseline. Further, participants in the control group will receive study monitors at the end of the study to reduce attrition and limit the use of PHMs during the study. Last, due to the small sample size, we will not have sufficient power to detect any group differences in the secondary outcomes. Therefore, any significant or nonsignificant findings must be interpreted with caution. We will, however, be able to observed trends and generate hypotheses to be tested in a fully powered study.

### Conclusion

This pilot feasibility study will provide the foundation for a larger trial that aims to improve sleep during pregnancy using a PHM device. The findings from this feasibility and acceptability pilot will provide valuable feedback on the design and implementation of the intervention.

## Abbreviations

ID: identification
PHM: personal health monitor
PSQI: Pittsburgh Sleep Quality Index

## References

[1] Facco FL, Kramer J, Ho KH, Zee PC, Grobman WA (2010). Sleep disturbances in pregnancy. Obstet Gynecol.

[2] Herring SJ, Nelson DB, Pien GW, Homko C, Goetzl LM, Davey A, Foster GD (2014). Objectively measured sleep duration and hyperglycemia in pregnancy. Sleep Med.

[3] Reutrakul S, Anothaisintawee T, Herring SJ, Balserak BI, Marc I, Thakkinstian A (2018). Short sleep duration and hyperglycemia in pregnancy: aggregate and individual patient data meta-analysis. Sleep Med Rev.

[4] Cai S, Tan S, Gluckman PD, Godfrey KM, Saw S, Teoh OH, Chong Y, Meaney MJ, Kramer MS, Gooley JJ, GUSTO Study Group (2017). Sleep quality and nocturnal sleep duration in pregnancy and risk of gestational diabetes mellitus. Sleep.

[5] Williams MA, Miller RS, Qiu C, Cripe SM, Gelaye B, Enquobahrie D (2010). Associations of early pregnancy sleep duration with trimester-specific blood pressures and hypertensive disorders in pregnancy. Sleep.

[6] Li R, Zhang J, Zhou R, Liu J, Dai Z, Liu D, Wang Y, Zhang H, Li Y, Zeng G (2017). Sleep disturbances during pregnancy are associated with cesarean delivery and preterm birth. J Matern Fetal Neonatal Med.

[7] Lee KA, Gay CL (2004). Sleep in late pregnancy predicts length of labor and type of delivery. Am J Obstet Gynecol.

[8] Abeysena C, Jayawardana P, Seneviratne RDA (2010). Effect of psychosocial stress and physical activity on low birthweight: a cohort study. J Obstet Gynaecol Res.

[9] Ahmed AH, Hui S, Crodian J, Plaut K, Haas D, Zhang L, Casey T (2018). Relationship between sleep quality, depression symptoms, and blood glucose in pregnant women. West J Nurs Res.

[10] Plancoulaine S, Flori S, Bat-Pitault F, Patural H, Lin J, Franco P (2017). Sleep trajectories among pregnant women and the impact on outcomes: a population-based cohort study. Matern Child Health J.

[11] Rasmussen MH, Strøm M, Wohlfahrt J, Videbech P, Melbye M (2017). Risk, treatment duration, and recurrence risk of postpartum affective disorder in women with no prior psychiatric history: a population-based cohort study. PLoS Med.

[12] Moline M, Broch L, Zak R (2004). Sleep problems across the life cycle in women. Curr Treat Options Neurol.

[13] Tsai S, Lee C, Wu W, Landis CA (2016). Sleep hygiene and sleep quality of third-trimester pregnant women. Res Nurs Health.

[14] Bazargan M (1996). Self-reported sleep disturbance among African-American elderly: the effects of depression, health status, exercise, and social support. Int J Aging Hum Dev.

[15] Baker JH, Rothenberger SD, Kline CE, Okun ML (2018). Exercise during early pregnancy is associated with greater sleep continuity. Behav Sleep Med.

[16] Kolu P, Raitanen J, Luoto R (2014). Physical activity and health-related quality of life during pregnancy: a secondary analysis of a cluster-randomised trial. Matern Child Health J.

[17] van Lee L, Chia A, Loy SL, Colega M, Tham EKH, Cai S, Yap F, Godfrey KM, Teoh OH, Goh D, Tan KH, Chong Y, Broekman BFP, Chong MFF (2017). Sleep and dietary patterns in pregnancy: findings from the GUSTO cohort. Int J Environ Res Public Health.

[18] Hollenbach D, Broker R, Herlehy S, Stuber K (2013). Non-pharmacological interventions for sleep quality and insomnia during pregnancy: a systematic review. J Can Chiropr Assoc.

[19] Omura JD, Carlson SA, Paul P, Watson KB, Fulton JE (2017). National physical activity surveillance: Users of wearable activity monitors as a potential data source. Prev Med Rep.

[20] Lyons EJ, Lewis ZH, Mayrsohn BG, Rowland JL (2014). Behavior change techniques implemented in electronic lifestyle activity monitors: a systematic content analysis. J Med Internet Res.

[21] Polzien KM, Jakicic JM, Tate DF, Otto AD (2007). The efficacy of a technology-based system in a short-term behavioral weight loss intervention. Obesity (Silver Spring).

[22] Pellegrini CA, Verba SD, Otto AD, Helsel DL, Davis KK, Jakicic JM (2012). The comparison of a technology-based system and an in-person behavioral weight loss intervention. Obesity (Silver Spring).

[23] Unick JL, O'Leary KC, Bond DS, Wing RR (2012). Physical activity enhancement to a behavioral weight loss program for severely obese individuals: a preliminary investigation. ISRN Obes.

[24] Wang JB, Cadmus-Bertram LA, Natarajan L, White MM, Madanat H, Nichols JF, Ayala GX, Pierce JP (2015). Wearable sensor/device (Fitbit One) and SMS text-messaging prompts to increase physical activity in overweight and obese adults: a randomized controlled trial. Telemed J E Health.

[25] Cadmus-Bertram LA, Marcus BH, Patterson RE, Parker BA, Morey BL (2015). Randomized trial of a Fitbit-Based physical activity intervention for women. Am J Prev Med.

[26] Thompson WG, Kuhle CL, Koepp GA, McCrady-Spitzer SK, Levine JA (2014). “Go4Life” exercise counseling, accelerometer feedback, and activity levels in older people. Arch Gerontol Geriatr.

[27] Random integer generator.

[28] August EM, Salihu HM, Biroscak BJ, Rahman S, Bruder K, Whiteman VE (2013). Systematic review on sleep disorders and obstetric outcomes: scope of current knowledge. Am J Perinatol.

[29] Michie S, Abraham C, Whittington C, McAteer J, Gupta S (2009). Effective techniques in healthy eating and physical activity interventions: a meta-regression. Health Psychol.

[30] Gilinsky AS, Dale H, Robinson C, Hughes AR, McInnes R, Lavallee D (2015). Efficacy of physical activity interventions in post-natal populations: systematic review, meta-analysis and content coding of behaviour change techniques. Health Psychol Rev.

[31] Mantua J, Gravel N, Spencer RMC (2016). Reliability of sleep measures from four personal health monitoring devices compared to research-based actigraphy and polysomnography. Sensors (Basel).

[32] IFTTT.

[33] Buysse DJ, Reynolds CF, Monk TH, Berman SR, Kupfer DJ (1989). The Pittsburgh Sleep Quality Index: a new instrument for psychiatric practice and research. Psychiatry Res.

[34] Qiu C, Gelaye B, Zhong Q, Enquobahrie DA, Frederick IO, Williams MA (2016). Construct validity and factor structure of the Pittsburgh Sleep Quality Index among pregnant women in a Pacific-Northwest cohort. Sleep Breath.

[35] Buysse DJ, Yu L, Moul DE, Germain A, Stover A, Dodds NE, Johnston KL, Shablesky-Cade MA, Pilkonis PA (2010). Development and validation of patient-reported outcome measures for sleep disturbance and sleep-related impairments. Sleep.

[36] Johns MW (1991). A new method for measuring daytime sleepiness: the Epworth sleepiness scale. Sleep.

[37] Baumgartel KL, Terhorst L, Conley YP, Roberts JM (2013). Psychometric evaluation of the Epworth sleepiness scale in an obstetric population. Sleep Med.

[38] Fatigue: a brief guide to the PROMISE Fatigue instruments.

[39] Schalet BD, Pilkonis PA, Yu L, Dodds N, Johnston KL, Yount S, Riley W, Cella D (2016). Clinical validity of PROMIS Depression, Anxiety, and Anger across diverse clinical samples. J Clin Epidemiol.

[40] Cox JL, Holden JM, Sagovsky R (1987). Detection of postnatal depression: development of the 10-item Edinburgh Postnatal Depression Scale. Br J Psychiatry.

[41] Kozinszky Z, Dudas RB (2015). Validation studies of the Edinburgh Postnatal Depression Scale for the antenatal period. J Affect Disord.

[42] Lean MEJ, Anderson AS, Morrison C, Currall J (2003). Evaluation of a dietary targets monitor. Eur J Clin Nutr.

[43] Saldana J (2016). The Coding Manual for Qualitative Researchers. 3rd Edition.

[44] Thomas E, Magilvy JK (2011). Qualitative rigor or research validity in qualitative research. J Spec Pediatr Nurs.

[45] Low CA, Bovbjerg DH, Ahrendt S, Choudry MH, Holtzman M, Jones HL, Pingpank JF, Ramalingam L, Zeh HJ, Zureikat AH, Bartlett DL (2018). Fitbit step counts during inpatient recovery from cancer surgery as a predictor of readmission. Ann Behav Med.

[46] Izmailova ES, Wagner JA, Perakslis ED (2018). Wearable devices in clinical trials: hype and hypothesis. Clin Pharmacol Ther.

[47] Shelgikar AV, Anderson PF, Stephens MR (2016). Sleep tracking, wearable technology, and opportunities for research and clinical care. Chest.

[48] Bianchi MT (2018). Sleep devices: wearables and nearables, informational and interventional, consumer and clinical. Metabolism.

[49] Anderson M (2017). Digital divide persists even as lower-income Americans make gains in tech adoption.

